# Puff Bars, Tobacco Policy Evasion, and Nicotine Dependence: Content Analysis of Tweets

**DOI:** 10.2196/27894

**Published:** 2022-03-25

**Authors:** Kar-Hai Chu, Tina B Hershey, Beth L Hoffman, Riley Wolynn, Jason B Colditz, Jaime E Sidani, Brian A Primack

**Affiliations:** 1 Graduate School of Public Health University of Pittsburgh Pittsburgh, PA United States; 2 School of Medicine University of Pittsburgh Pittsburgh, PA United States; 3 College of Education and Health Professions University of Arkansas Fayetteville, AR United States

**Keywords:** tobacco, policy, social media, e-cigarette, twitter, mHealth, dependence, addiction, nicotine

## Abstract

**Background:**

Puff Bars are e-cigarettes that continued marketing flavored products by exploiting the US Food and Drug Administration exemption for disposable devices.

**Objective:**

This study aimed to examine discussions related to Puff Bar on Twitter to identify tobacco regulation and policy themes as well as unanticipated outcomes of regulatory loopholes.

**Methods:**

Of 8519 original tweets related to Puff Bar collected from July 13, 2020, to August 13, 2020, a random 20% subsample (n=2661) was selected for qualitative coding of topics related to nicotine dependence and tobacco policy.

**Results:**

Of the human-coded tweets, 2123 (80.2%) were coded as relevant to Puff Bar as the main topic. Of those tweets, 698 (32.9%) discussed tobacco policy, including flavors (n=320, 45.9%), regulations (n=124, 17.8%), purchases (n=117, 16.8%), and other products (n=110, 15.8%). Approximately 22% (n=480) of the tweets referenced dependence, including lack of access (n=273, 56.9%), appetite suppression (n=59, 12.3%), frequent use (n=47, 9.8%), and self-reported dependence (n=110, 22.9%).

**Conclusions:**

This study adds to the growing evidence base that the US Food and Drug Administration ban of e-cigarette flavors did not reduce interest, but rather shifted the discussion to brands utilizing a loophole that allowed flavored products to continue to be sold in disposable devices. Until comprehensive tobacco policy legislation is developed, new products or loopholes will continue to supply nicotine demand.

## Introduction

From 2011 to 2019, current e-cigarette use among US high school students increased from 1.5% to 27.5% [1], prompting the US Surgeon General to declare a youth vaping epidemic. An appealing aspect of e-cigarette use to adolescents was the availability of flavors [2]. Among this population, initial use of flavored e-cigarettes is associated with progression to current e-cigarette use [3].

Driven by the results of the 2019 National Youth Tobacco Survey (NYTS) and other reports of increased use of tobacco products by youth, the US Food and Drug Administration (FDA) took action to address this epidemic in December 2019 by raising the federal minimum age for sale of tobacco products (including e-cigarettes) from 18 to 21 years and by prioritizing enforcement against illegal flavored (eg, fruits) e-cigarettes [4,5]. Likewise, many US states have enacted legislation to restrict flavored e-cigarettes [6]. In an effort to balance considerations for adult smokers trying to quit cigarettes, the federal flavor ban was focused on cartridge-based products, such as those sold by JUUL (JUUL Labs)—at the time, the device used by a majority of youth who were current e-cigarette users [1].

**Figure 1:**
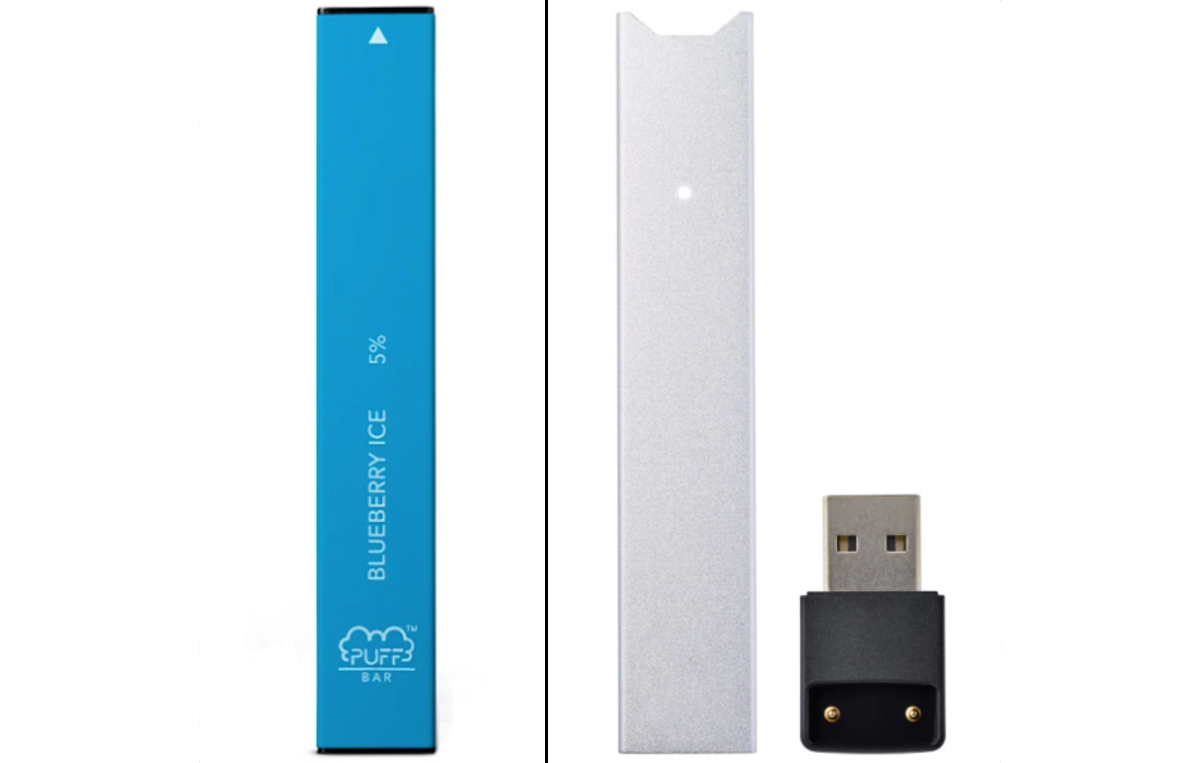
Puff Bar and JUUL comparison. Left: Puff Bar device; right: JUUL device.

However, the actions by the FDA may have resulted in unintended consequences. In the case of e-cigarette–related policy, loopholes allowed for disposable devices such as Puff Bar to continue to be sold, even in prohibited flavors. Puff Bars are single-use, disposable, flavored e-cigarette products. The design and packaging of Puff Bar are similar to those of JUUL (Figure 1). Puff Bar e-cigarettes come in 25 different flavors (eg, strawberry banana). There is evidence that Puff Bar is targeting its products and advertisements to youth. For example, the company produced flavor pods (Puff Krush) that are advertised as an “add-on” for JUUL pods following JUUL’s removal of most flavors from the US market, which were extremely popular with youth [7]. In 2020, the NYTS reported that the use of disposable e-cigarettes among current high school users increased by approximately 1000% from 2019 [8].

On July 13, 2020, Puff Bar announced that they were ceasing online sales in the United States [9]. One week later, the FDA announced that it issued warning letters to 10 companies, including Cool Clouds Distribution, Inc (Puff Bar’s parent company), to remove their products from the market, citing their introduction after the 2016 deeming rule bringing all tobacco products under the authority of the FDA [10]. Puff Bar was also cited for marketing their product as a modified risk tobacco product without FDA approval [10]. However, evidence suggests that Puff Bar sales were continuing despite the FDA warnings. For example, at the time this article was written, the Puff Bar website still had a “store locator” function [11] listing retailers across the US.

The continued sales of flavored products in disposable devices may be contributing to youth use of products containing high levels of nicotine. In contrast to contemporary products that contain freebase nicotine, Puff Bar e-cigarettes contain nicotine salt formulations (similar to JUUL) that deliver nicotine in a quickly metabolized and palatable manner, with nicotine concentrations as high as 5% [12,13]. Research indicates that users of JUUL’s higher nicotine level products (ie, 5%) experience symptoms of dependence and acute nicotine effects [14]. Likewise, nicotine dependence in past-month adolescent e-cigarette users is significantly associated with increased nicotine concentrations [15]. Thus, despite the intended goal of reducing youth tobacco use through legislative and policy activities, unintended loopholes allowed youth to access the same products, just in a different form.

**Figure 2:**
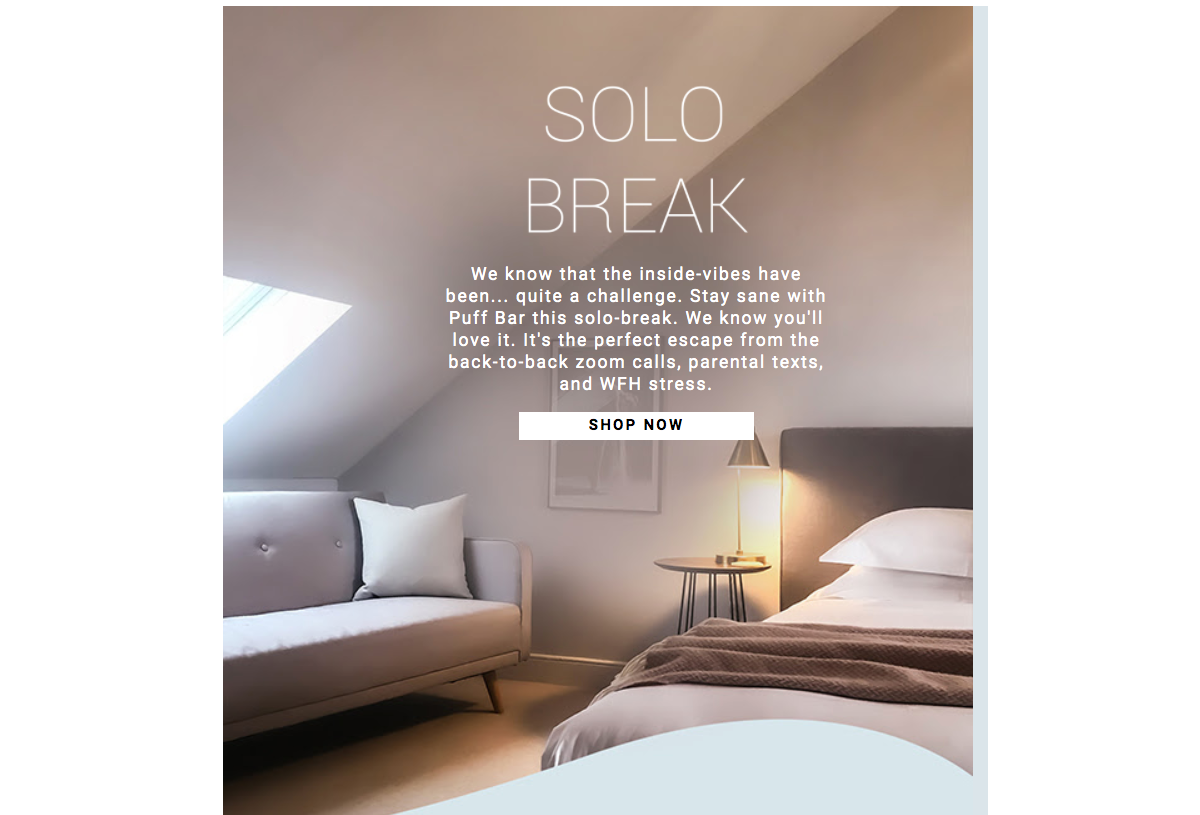
Puff Bar advertisement during global pandemic.

Research has consistently found that e-cigarette discussions on social media platforms can quickly diffuse marketing messages, activate brand awareness, and reach large numbers of adolescents [16-18]. An analysis of videos on TikTok, a social media platform particularly popular with younger populations [19], found that the 10 most popular videos depicting Puff Bar were viewed between 2.8 and 42.4 million times and that 2 of the videos depicted clear youth-related content (eg, an underage youth purchasing a Puff Bar) [20]. Additionally, during the COVID-19 pandemic social distancing restrictions, Puff Bar released an advertisement picturing a bedroom and suggested that their products would allow for escape from “back-to-back zoom calls, parental texts, and WFH [work from home] stress” (Figure 2) [21].

With growing risk of adolescent use of disposable e-cigarette devices, studying Puff Bar on social media may offer insight into topics of discussion and trends that are emerging [22]. Twitter has become a valuable source of publicly observable data for public health practitioners to better understand attitudes toward e-cigarette use, advertisements targeted to youth, and discussions of tobacco regulations [23-26]. Pew Research has also found that Twitter users tend to be younger than the overall US adult population [27]. Specifically, Puff Bar-related discussions on Twitter may give insight into how these products are being used as an alternative to products that fall under federal and state restrictions. Evaluating the potential impact of legal actions related to e-cigarettes will provide important information to the public health community. Thus, this study sought to examine Puff Bar-related discussions on Twitter to identify themes related to tobacco policy as well as unanticipated outcomes of legal and regulatory loopholes, such as effects from continued use of flavored, high nicotine concentration salt formulations, for use in future research.

## Methods

### Sample Selection

We used the Real-time Infoveillance of Twitter Health Messages software framework to collect Twitter posts (ie, tweets) containing the terms “puffbar” or “puff bar” for 1 month from July 13, 2020, to August 13, 2020 [28]. The Real-time Infoveillance of Twitter Health Messages allows for real time collection of all publicly available tweets matching a specified set of keywords through Twitter’s filtered data stream application programming interface. This start date aligned with the date Puff Bar announced ceasing online sales in the United States [9]. This resulted in 13,304 tweets, of which 4785 (36%) were retweets and 8519 (64%) were original tweets.

We obtained a 20% random subsample of original tweets (n=2661) for human coding; this process has previously demonstrated to be both feasible for human qualitative coding and generalizable to the full data set [28].

### Ethical Approval

The University of Pittsburgh Institutional Review Board (IRB) determined that the proposed activity is not research involving human subjects as defined by DHHS and FDA regulations (STUDY19080214).

### Codebook Development

We developed a codebook based on a hybrid process that included consideration of our research question, prior analysis of e-cigarette discourse on Twitter [14,29], tobacco policy, and an inductive analysis of 100 relevant tweets that were not included in the final sample. First, we included a code for relevance to the research topic of Puff Bar-related discussions (relevant). Tweets that did not contain the disposable e-cigarette Puff Bar as the main topic (eg, “I go to my car to get my puff bar and somebody left me a rose on my car. I can’t stop smiling!”*)* were deemed not relevant.

Relevant tweets were coded for topics informed by 2 areas of research, which were nicotine dependence and tobacco policy. Topics for nicotine dependence included language that suggests dependence on Puff Bar and signs of nicotine addiction or withdrawal related to Puff Bar use. These categories were informed by prior research that examined similar content posted about JUUL [14]. Topics for tobacco policy included whether posted content were commercially marketing Puff Bar or a business selling Puff Bar, references to purchasing a Puff Bar, references to underage use of Puff Bar, regulations of Puff Bar, price of Puff Bar, references to Puff Bar flavor, and references to other e-cigarette products. The codebook was validated through analysis of 100 relevant tweets that were not included in the final sample by 2 experienced Twitter coders. Following this, the final codebook was codified, presenting clear definitions and examples for each code (Table 1).

**Table 1:** Definitions for categorical codes and example tweets. Examples are paraphrased.

Code and subcode		Definition	Examples
**Tobacco policy**		Puff Bar in relationship to laws or regulations that could affect use of Puff Bar	
	Commercial	Marketing for Puff Bar or shops selling Puff Bar	Check out what our customers are saying about Puff Bar Disposable Pod device!
	Black market	Reference to illegal purchase of a Puff Bar or buying a knockoff	Yo the puff bar black market is wild I hope the govt knows they created both the fake puff bar prob & the fake thc oil problems all by themselves
	Buying	Concretely obtaining or trying to obtain Puff Bar	Can I get a puffbar on Instacart? Got a free puff bar at the gas station :)
	Underage use	Underage use (ie, under 21) of Puff Bar	I wish I could explain to you guys how flabbergasted I am to have just met a 5 year old child 2/ a puff bar…he talked about disposable vapes for 5 minutes & rated various flavors This little boy really asked if I could buy him a puff bar and when I did, he went goat and then says I can’t get it anymore I’m sorry
	Regulations	Regulations on Puff Bar (cannot buy, cannot access, etc)	Why do I gotta be 21 to buy myself a puff bar FDA calls for removal of fruity and disposable Puff Bar vapes devices
	Price	Price of a Puff Bar	Anyways does anyone wanna paypal me 16 dollar so I can buy a fucking puff bar Maaan I shouldn’t have hit ur puff bar bc now my ass is spending $15-20 every other week on nic
	Flavors	Flavors of Puff Bar	Strawberry banana puff bar is just vaping a gogurt Peach ice and lychee seemed to be the favorite/most popular flavors
	Other products	Other vaping products, including Puff XXL or JUUL	Checkout the newest Puff XXL 1600 disposable device Why did nobody tell me that air bars get me more puffs than a puff bar plus they r cheaper
References to COVID-19		Use of Puff Bar during the pandemic	I hate when my puff bar starts spittng at me. C’mon bitch were in a pandemic!
**Dependence**		Puff Bar in association with words that specify dependence on Puff Bar	
	Puff bar as a meal	Puff Bar as a meal or in place of food, using terms such as meal, breakfast, lunch, or dinner	My power meal today: one puff bar plus an energy drink Had a whole lychee puff bar as all of my meals today. I’m thriving babeee
	Losing access	Not being able to use Puff Bar to an external factor such as losing it or the battery dying	On 4 hour drive and my puff bar completely died I lost my puff bar for an hour and found it in my bra. Whoops
	Self-report	Self-report of being addicted or dependent on Puff Bar	Just tried a puff bar and I’m definitely addicted Bought myself a puff bar. Time to bring back my nicotine addiction
	Does not last	Using up Puff Bar quickly	I’ve never been able to make a puff bar last more than 48 hours Another day. Time for another puff bar
Acute nicotine effects		Puff Bar in association with words that specify acute nicotine effects	My puff bar got me buzzing like a bee That puffbar feeling - lightheadedness
Quitting or withdrawal		Quitting Puff Bar or experiencing signs of nicotine withdrawal from lack of Puff Bar	I have a problem, so after this puff bar runs out, I'm NOT buying another one Only positive of being at my parents house is that i finally quit my puffbar quarantine habit

### Coding Procedures

The tweets were coded by 2 independent individuals, with adjudication of disagreements by a supervising researcher. The coders were provided with the tweet text and a URL link to each tweet. During the coding process, all relevant tweets that were publicly available at the time of coding were viewed on Twitter so that visuals in the tweet (eg, images, videos, and emoji) could also be assessed. The text from unavailable tweets was still included in the coding and thematic analysis to preserve comprehensiveness of the original data. The codes were not mutually exclusive.

We calculated interrater reliability using the Cohen κ, and disagreements were adjudicated between the 2 coders. In instances where the coders could not reach consensus, the lead author had final determination. After 4 rounds of independent double coding (100 tweets each round), interrater reliability was considered good to excellent (Cohen κ=0.71-1.00) for all categories [30]. The remaining tweets were split between the 2 coders for independent coding.

### Analysis

We calculated descriptive statistics for each coding category and used a thematic content analysis approach to analyze qualitative data [31]. To conduct the content analysis, the coders wrote annotations and memos throughout the coding process and highlighted specific words or phrases within tweets that exemplified the themes. The coders then met with supervising researchers to synthesize themes with salient examples from the observed data.

This study was approved by the University of Pittsburgh Institutional Review Board. To protect tweeters from identification, all examples provided in the text and tables are paraphrased versions of original tweets.

## Results

Of the random sample of tweets (n=2661), 80.2% (n=2123) were coded as relevant to the research question. Of these relevant tweets, 698 (32.9%) tweets discussed topics relevant to tobacco policy (Table 2). In table 2, the percentages for subcodes are presented as the percent of tweets within total tweets for that code.

**Table 2:** Frequencies of coding categories for relevant tweets (n=2123). Categories are not mutually exclusive; therefore, proportions will not always add up to 100%.

Code and subcode		Frequency, n (%)
**Tobacco policy**		698 (32.9)
	Flavors	320 (45.9)
	Regulations	124 (17.8)
	Buying	117 (16.8)
	Other products	110 (15.8)
	Black market	31 (4.4)
	Price	36 (5.2)
	Underage use	24 (3.4)
	Commercial	20 (2.9)
**Dependence**		480 (22.6)
	Losing access	273 (56.9)
	Self-report	110 (22.9)
	Puff bar as a meal	59 (12.3)
	Does not last	47 (9.8)
Quitting or withdrawal		52 (2.4)
Acute nicotine effects		50 (2.4)
References to COVID-19		11 (0.5)

The most frequent themes relevant to tobacco policy were references to Puff Bar flavors (n=320, 45.9%; eg, “I bought a peach ice puff bar and it is so yummy”). There was a similar prevalence of references to buying Puff Bar (n=117, 16.8%; eg, “setting my alarm to go the puff bar store tomorrow morning”) and other products (n=110, 15.8%; eg, “a puff bar only lasts me a day so switched to viva. Last 3+ days”).

Approximately 22% (n=480) of the tweets referenced dependence in the context of Puff Bar. A majority of these tweets referenced losing access to Puff Bar (n=273, 56.9%; eg, “on a long drive and my puff bar died. Send help. Im so upset”). Another theme was users tweeting about their Puff Bar lasting less than 48 hours due to frequent use (n=47, 9.8%; eg, “I wonder why I can’t breathe yet I go through a puff bar in 2 days”). Additionally, approximately a quarter (n=110, 22.9%) of tweets referencing dependence involved the user self-reporting dependence on Puff Bar (eg, “I need a puff bar so bad, but I will stay strong. I want to get rid of this mf addiction”).

Users also reported acute nicotine effects, such as feeling a buzz or high when using Puff Bar (n=50, 2.4%; eg, “this puff bar lightheadedness feels so good”). Users reported other symptoms related to nicotine, such as headaches (eg, “is my headache a sign of coronavirus or my puff bar addiction?”) and upset stomach (eg, “I hit my puff bar until my stomach hurts everyday haha”).

## Discussion

### Principal Findings

This study examined Puff Bar-related discussions on Twitter to identify themes related to tobacco policy and dependence. Despite federal regulations and FDA warnings against Puff Bar, the results of this study suggest that the purchasing and use of Puff Bar products are still being discussed on social media. Discussions of Puff Bar flavors were prevalent, accounting for a plurality (45.9%) of tweets classified as relevant to tobacco policy; these products are circumventing a federal ban designed to protect youth from the appeal of nicotine products. Similarly, the second most common theme was focused on regulations (n=124, 17.8%). As a result, the outcome of the FDA ban of e-cigarette flavors did not reduce demand, but rather shifted more attention to different e-cigarette brands that utilize a loophole for disposable devices.

We found similar dependence and acute nicotine effect themes as prior research on JUUL products and cigarettes, such as self-report of dependence, frustration over losing access to the device, compulsive use, and self-reports of physical effects of nicotine exposure including suppression of appetite [14,32]. Again, this suggests that despite legislative and policy activities to reduce youth access to and use of flavored, high nicotine-containing and dependence-forming products, they continued to be available due to exploitation of loopholes. The fact that high school students’ use of disposable e-cigarettes such as Puff Bar increased by 1000% from 2019 to 2020 is evidence that these loopholes can contribute to continued use of e-cigarettes by youth.

Due to new federal tobacco policies, discussions about Puff Bar offer a view into topics relevant to tobacco policy including e-cigarette regulations and how they might affect access to Puff Bar (17.9%) and buying Puff Bar (16.8%). The results suggest that while the legality of Puff Bar sales might be recognized and discussed by Twitter users, people continue to purchase them even though the products are banned in the United States. This is especially alarming considering that commercial content (ie, posts by retailers) were very low (2.9% of tobacco policy tweets, 0.9% of all relevant tweets) when compared with other e-cigarette research [33,34].

The results of this study align with data from the NYTS that demonstrate a shift to disposable e-cigarettes following FDA tobacco policies. While the federal flavor ban was enacted to curb youth e-cigarette use, it is unfortunate that exemptions created loopholes to allow continued access to flavored products. While outside the scope of this study, future research should consider identifying what types of accounts are posting this content. Specifically, understanding whether commercial tweets are being posted by Puff Bar users, commercial vendors, news organizations, or others could help to better inform counter messaging or preventive measures. Additionally, it would be valuable for researchers to be guided by a framework that can address both legal and health concerns. For example, legal epidemiology, the scientific study and deployment of law as a factor in the cause, distribution, and prevention of disease and injury in a population, might inform stronger and more comprehensive policies [35]. Legal epidemiology involves the three following components: (1) legal prevention and control—the study and application of laws and legal practices as interventions to prevent disease and injury and as enablers of effective public health administration; (2) legal etiology—the study of laws and legal practices as causes of disease and injury; and (3) policy surveillance—the ongoing, systematic collection, analysis, and dissemination of information about laws and other policies of importance to health [36]. In combination with continued real time infoveillance of e-cigarette discussions, this framework could help better understand how tobacco policy (eg, age or flavor restrictions) and implementation can affect health outcomes.

Puff Bar acknowledged that their products were excluded from the FDA regulation banning most flavors. On February 21, 2020, Puff Bar posted on their official blog a lament about federal regulations “determined to eliminate vaping as a whole” stating that “disposable devices like Puff Bars and e-liquid used in refillable tank systems can still carry flavors like fruits and desserts” [37]. These, and other similar arguments, have been used by JUUL in order to continue promoting their products, particularly through advertisements targeted to adolescents [38]. Puff Bars—which have now resumed sales—have become the latest device to replace previously banned tobacco products. Until we are able to develop comprehensive tobacco policy, new products that capitalize on policy loopholes will continue to supply the demand for nicotine, particularly by adolescents through targeted advertisements or flavored products. We suggest that a framework such as legal epidemiology could offer a unique lens to understand the confluence of tobacco policy and health outcomes, helping inform policy makers to better understand and strengthen the practical implications of tobacco policies.

### Limitations

The results of this study should be considered in the context of the following limitations. Twitter users are not representative of the general population, although it is frequently used by adolescents and young adults [27], a population that also frequently uses e-cigarettes. While interpretation of tweets using qualitative analysis can be subjective, we minimized subjectivity by using a systematic coding procedure and the use of experienced Twitter coders; nonetheless, these tweets are discussion of purchasing, rather than the act itself. We also did not use location data; thus, there is the possibility that some tweets are by non-US users. Finally, our results are constrained by the keywords and time period used in our search parameters. Future research could expand these parameters to widen the scope of the investigation.

### Contribution to Literature

Here, we summarize the key findings of our research: (1) laws and regulations around e-cigarettes are rapidly changing in response to increased concern about the use of the products by youth, and prior research suggests the focus of flavor bans on devices such as JUUL may have created a policy loophole that was filled by disposable devices such as Puff Bar; (2) however, it is not yet known how Puff Bar is being used as an alternative to traditional e-cigarettes that fall under federal and state restrictions; (3) our analysis of tweets related to Puff Bar suggests that the FDA ban of e-cigarette flavors did not reduce interest, but rather shifted the discussion to brands utilizing a loophole for disposable devices and suggests the importance of using a framework such as legal epidemiology when researching and evaluating tobacco policy.

### Conclusion

This study found similar dependence and acute nicotine effect themes in Puff Bar-related discussions on Twitter compared to prior research on JUUL and cigarettes [14]. We also found that discussions about Puff Bar on Twitter provided insight into topics relevant to tobacco policy, including flavors, e-cigarette regulations, and purchasing Puff Bar. Our results, in conjunction with evidence by the NYTS and other data sources, suggest that the FDA ban of e-cigarette flavors did not reduce demand, but rather shifted the supply to brands utilizing a loophole for disposable devices. Until comprehensive tobacco policy legislation is developed, new products or loopholes will continue to supply nicotine demand.

## Abbreviations

FDA: Food and Drug Administration
NYTS: National Youth Tobacco Survey
